# The Crucial Roles of Phospholipids in Aging and Lifespan Regulation

**DOI:** 10.3389/fphys.2021.775648

**Published:** 2021-11-23

**Authors:** Yucan Dai, Haiqing Tang, Shanshan Pang

**Affiliations:** School of Life Sciences, Chongqing University, Chongqing, China

**Keywords:** membrane lipid, aging, phospholipid, lifespan, organelle

## Abstract

Phospholipids are major membrane lipids that consist of lipid bilayers. This basic cellular structure acts as a barrier to protect the cell against various environmental insults and more importantly, enables multiple cellular processes to occur in subcellular compartments. Numerous studies have linked the complexity of membrane lipids to signal transductions, organelle functions, as well as physiological processes, and human diseases. Recently, crucial roles for membrane lipids in the aging process are beginning to emerge. In this study, we summarized current advances in our understanding of the relationship between membrane lipids and aging with an emphasis on phospholipid species. We surveyed how major phospholipid species change with age in different organisms and tissues, and some common patterns of membrane lipid change during aging were proposed. Further, the functions of different phospholipid molecules in regulating healthspan and lifespan, as well as their potential mechanisms of action, were also discussed.

## Introduction

The relationship between lipids and aging has been well recognized. The contents, composition, and metabolism of fatty acid (FA) are altered in aged or long-lived humans and model organisms (Papsdorf and Brunet, 2019). Moreover, studies in model organisms such as *Caenorhabditis elegans* have revealed that various FA species could extend lifespan when supplemented in the diet, which includes monosaturated oleic acid, palmitoleic acid, cis-vaccenic acid, and oleoylethanolamine, as well as polyunsaturated a-linolenic acid, arachidonic acid, and dihomo-g-linolenic acid (Goudeau et al., 2011; Rourke et al., 2013; Folick et al., 2015; Han et al., 2017; Qi et al., 2017). These unsaturated FAs function mainly through classic longevity factors, such as DAF-16/FOXO3, SKN-1/Nrf2, and HSF-1/HSF1, to regulate healthspan and lifespan (Labbadia et al., 2015; Steinbaugh et al., 2015; Papsdorf and Brunet, 2019).

Despite these advances linking FA to longevity regulation, little is known about their mechanisms of action. Generally, FAs function through several major mechanisms, including signaling molecules, energy resources, substrates for post-translational modifications, and the components of complex lipids (Van Meer et al., 2008; Shimizu, 2009; Nakamura et al., 2014; Saliba et al., 2015; Resh, 2016; Harayama and Riezman, 2018). Take oleoylethanolamine for example, it acts as a signaling molecule and regulates animal lifespan by direct binding and activation of the nuclear hormone receptor NHR-80 (Folick et al., 2015). But to date, only a few FAs were found to exert their functions directly as signaling molecules, or as substrates for post-translational modifications. The majority of FAs are incorporated into complex lipids such as membrane lipids as their acyl chains, thus affecting the structure, composition, and function of the membrane (Van Meer et al., 2008; Sezgin et al., 2017; Harayama and Riezman, 2018). Therefore, it is conceivable that FAs may regulate lifespan by acting as the important components of membrane lipids, potentially linking membrane homeostasis to lifespan regulation.

Membrane lipids, mainly phospholipids (PLs; also known as glycerophospholipids), consist of the lipid bilayer that acts as barriers between the cell and environment, and between different cellular compartments. However, numerous studies suggest that the lipid bilayer not only function as structural barriers but also play crucial roles in the regulation of multiple cellular processes (Shimizu, 2009; Wu et al., 2016; Sunshine and Iruela-Arispe, 2017; Harayama and Riezman, 2018). Also, this idea is supported by the diversity of membrane lipids (different membrane lipid species and different acyl chains within certain membrane lipids) (Hishikawa et al., 2014; Antonny et al., 2015), which is far more beyond the need for barrier function. In regard to the aging process, studies in several model organisms have reported the association of the contents and compositions of many membrane lipids with animal age (Papsdorf and Brunet, 2019), supporting potential roles for the membrane lipids in aging modulation. In this review, we focused on PLs and summarized recent advances that link PL homeostasis to the aging process and discussed their potential mechanisms of action. Other membrane lipids such as sphingolipids were not discussed in the current review.

## Phospholipid Metabolism and Diversity

Phospholipids are major structural lipids of the eukaryotic membrane, including phosphatidylcholine (PC), phosphatidylethanolamine (PE), phosphatidylserine (PS), phosphatidylinositol (PI), phosphatidic acid (PA), and cardiolipin (CL). These major PL species share similar structures containing two FAs attached to the *sn*-1 and *sn*-2 positions and a different phosphate headgroup at the *sn*-3 position of the glycerol backbone. The different compositions of these PLs may account for the membrane diversity of various subcellular compartments and thus the functions of the organelles. Take PC as an example, it is the most abundant PLs that account for over 50% of total PLs in the cell. It mainly resides in the endoplasmic reticulum (ER), the site of membrane lipid biosynthesis, but its contents are relatively less in the plasma membrane (Van Meer et al., 2008). The maintenance of PC homeostasis is critical for the organellar function, while the reduction of PC represents cellular stress known as lipid bilayer stress (Volmer et al., 2013; Halbleib et al., 2017; Shyu et al., 2019). Therefore, the cell evolves an elegant adaptive mechanism to survey the PC content and the loss of PC has been found to affect multiple cellular processes through this stress-responsive pathway (Koh et al., 2018; Ho et al., 2020). Another extreme example of the diversity of PL composition is organelle-specific PLs. CL is a mitochondria-specific membrane lipid, whose content has been found to have great impacts on mitochondrial functions and to be related to various mitochondrial diseases (Chicco and Sparagna, 2007; Houtkooper and Vaz, 2008; Schlame, 2008; Pizzuto and Pelegrin, 2020).

The acyl chain composition also accounts for the diversity of PLs, which differ largely in the lengths of the chains and the numbers and positions of the double bonds. These chemical variations may affect the membrane protein-lipid interactions and therefore the cellular signaling properties of the membrane proteins (Antonny et al., 2015; Saliba et al., 2015; Wu et al., 2016; Harayama and Riezman, 2018). In addition, PLs containing the unsaturated acyl chains are more fluidic than the saturated ones and thus the overall degree of the FA unsaturation in PLs could affect the membrane fluidity, which has been found to regulate numerous signaling pathways, cellular processes, and human diseases (Los and Murata, 2004; D’Auria and Bongarzone, 2016; Ammendolia et al., 2021). Therefore, as for the aging study, it is important to understand how the contents and compositions of PLs interact with proteins in longevity pathways and how such interactions determine the healthspan and lifespan. In the following section, we will discuss the relationship between aging and essential PLs.

## Phosphatidylcholine

### Association Between Phosphatidylcholine and Age

Multiple studies have reported the changes of PC contents as animal ages, with species and tissue specificity. For example, it was reported that the contents of most PCs are notably decreased in the aged nematodes (Gao et al., 2017; Wan et al., 2019). Additionally, the overall PC contents show a significant reduction in the kidney of aged mouse (Braun et al., 2016), while the accumulation of PCs has been detected in the hippocampus of a brain aging mice model (Lin et al., 2016). In line with this, major mitochondrial PC species are found to be increased in the brain of aged humans (Hancock et al., 2015, 2017). Thus, the reduction of overall PC contents appears to be a general feature of animal aging, except for the aged brain where the PC contents seem to be increased. Studies in long-lived organisms further support this idea, as overall PC contents are greater in multiple tissues of the long-lived naked mole-rat compared with that of mice (Mitchell et al., 2007). It is also the case for humans, as most PC species are higher in the centenarians than that in the elderly (Montoliu et al., 2014).

More intriguingly, besides the accumulation of overall PC contents, the long-lived centenarians have a unique metabolic signature of PC, showing that several specific PC species are unexpectedly decreased (Collino et al., 2013). These decreased PCs remain similar between the centenarians and young people, while elevating in the elderly (Collino et al., 2013), thus may representing a youth or longevity signature. A study in model organism *C. elegans* also shows that certain PCs, mainly some unsaturated ones, remain at low levels in long-lived mutants regardless of age (Wan et al., 2019). The long-lived naked mole rats also have low levels of PCs that contain unsaturated docosahexaenoic acid (DHA, 22:6) as acyl chains, compared with mice. As such, we propose that specific PCs, most likely some unsaturated ones, are negatively associated with animal longevity.

### Lifespan Regulation by Phosphatidylcholine

It seems that the overall PC contents and specific PCs are associated with the age difference. In *C. elegans*, a Dietary supplement of PC mixture can significantly extend the lifespan and improve the healthspan of nematodes via the longevity transcription factor DAF-16 (Kim et al., 2019), which is consistent with the idea that the decline of total PCs is not only a biomarker of aging but also a reason for it. The mechanism underlying the total PCs effects on lifespan is currently not clear but may be related to membrane fluidity. The decline of total PCs can cause membrane stiffening (Dawaliby et al., 2016) that is observed in aged cells and animals (Yu et al., 1992; Levi et al., 1997). Moreover, this age-associated decline of membrane fluidity can be reversed by longevity-inducing interventions like dietary restriction (Yu et al., 1992).

Unsaturated PC species may act differently with total PCs in the context of lifespan regulation. A recent *C. elegans* study reported that the levels of unsaturated PCs are decreased in a long-lived *C. elegans* model. More importantly, the reduction of unsaturated PC species likely function in the endoplasmic reticulum (ER) to promote longevity by activating the ER-resident calcium channel, downstream calcium-sensitive kinases, and the transcription factor DAF-16 (He et al., 2021), suggesting that specific unsaturated PCs may interact with membrane proteins to regulate longevity. The finding that reduction of unsaturated PC is beneficial for lifespan extension is consistent with the idea that low contents of unsaturated PCs may be a longevity signature. Understanding how the changes of certain unsaturated PCs determine lifespan deserves further investigation.

### Phosphatidylcholine and Brain Aging

Aging is a complex process associated with the functional decline of essential organs and tissues, thus representing a major risk factor for many chronic diseases referred to as aging-related diseases. As PCs accumulate in the brain of aged animals (Hancock et al., 2015, 2017), studies also pay attention to the function of PCs in neurodegenerative diseases such as Alzheimer’s disease (AD). In patients with AD, the contents of unsaturated PC 36:5 and PC 38:6 are lower in the plasma and are correlated with hippocampus atrophy (Kim et al., 2017). Consistently, the higher contents of several unsaturated PCs are associated with a slower decline in AD-related cognition (Li et al., 2019). By contrast, the accumulation of PC-O (16:0/2:0) is found to associate with multiple AD pathologies, such as the aggravated tau pathology, enhanced vesicular release, and signaling neuronal loss (Bennett et al., 2013). Thus, it appears that certain unsaturated PCs are critical for the functional maintenance of neurons.

It is not fully understood how certain PCs maintain neuronal function and protect against neurodegenerative diseases. A key and common pathology of neurodegenerative diseases is protein aggregation. The ER is critical for protein folding. when misfolded proteins accumulate in the ER (also known as ER stress), ER unfolded protein response is induced to assist protein folding and maintain cellular proteostasis. Indeed, PC disturbance has been linked to abnormal ER function and protein aggregation (O’Leary et al., 2018; Kumar et al., 2019; Shyu et al., 2019). As a consequence, PC disturbance is recognized as a stress to the ER and can promote the ER UPR (Volmer et al., 2013; Halbleib et al., 2017; Ho et al., 2020). A recent study of *C. elegans* also supports the crucial of certain PCs in ER homeostasis. The contents of unsaturated PC are increased in the somatic cells of *C. elegans* upon DNA damage, which represents an aging risk factor. Moreover, the elevation of unsaturated PCs is critical for promoting the ER UPR without causing ER stress, thus enhancing cellular maintenance (Deng et al., 2021). Intriguingly, the neurons and *C. elegans* somatic cells share some common features, as they are both postmitotic cells that cannot proliferate and are irreplaceable. In this regard, we propose that certain unsaturated PC species may be of great importance for the functional maintenance of postmitotic cells such as neurons during the aging process.

## Phosphatidylethanolamine

### Association Between Phosphatidylethanolamine and Age

Phosphatidylethanolamine is the second abundant PL in organisms. Emerging pieces of evidence show that PE contents also change with age. Major PE species decrease significantly in the aged nematodes (Gao et al., 2017), as well as in the brain and kidney of old mice (Braun et al., 2016; Lin et al., 2016), suggesting that PE reduction may also be a general feature of aging. Moreover, a human lipidomic study has found that the contents of ether lipid derived from PE are lower in the centenarians compared with that in the elderly people (Pradas et al., 2019), suggesting this specific form of PE may function as a centenarian signature that may promote longevity. Interestingly, although the contents of overall mitochondrial PE in the brains of the elderly are reduced, mitochondrial PEs containing unsaturated docosahexaenoic acid (DHA, 22:6) were found to be increased significantly (Hancock et al., 2015, 2017). Due to the importance of DHA for neuronal development and function, the increase in DHA-containing PEs with age may be an adaptive and protective mechanism for brain aging, suggesting specific PE species and overall PE contents may affect health during aging differentially.

### Lifespan Regulation by Phosphatidylethanolamine

Studies has shown that the increase of PE contents, either by dietary supplementation of PE precursor ethanolamine or by the overexpression of PE-biosynthetic enzymes phosphatidylserine decarboxylases (PSD), extends lifespan in model organisms of yeast, fly, and mammals (Rockenfeller et al., 2015). And the lifespan extension effects of PE are associated with increased autophagic flux (Rockenfeller et al., 2015), a positive lifespan regulator in many model organisms (Hansen et al., 2018), suggesting PE supplementation may increase lifespan by promoting autophagy.

In contrast, the reduction of PE by suppressing PSD causes ROS production and accelerates aging in yeast (Rockenfeller et al., 2015). The relationship between PE and ROS is also supported by a *C. elegans* study that supplementation of PE enhances the resistance to oxidative stress and promotes longevity via DAF-16 (Park et al., 2021). These findings suggest that PE plays pivotal roles in life extension, likely acting as a regulator of ROS production. ROS is a byproduct of mitochondrial respiration and is generally associated with mitochondrial defects or dysfunction. Indeed, PSD-mediated PE production occurs in the mitochondrial inner membrane and is found to be essential for the activity of the electron transport chain (Calzada et al., 2019). Accordingly, PSD knockdown compromised the mitochondrial integrity and muscle mass in the mice (Selathurai et al., 2019). Thus, it is conceivable that mitochondria activity is key for lifespan determination in response to PE alteration.

## Phosphatidylserine

The contents of major PS species are also found to decrease during aging (Lin et al., 2016; Šmidák et al., 2017). Consistently, supplementation of PS shows beneficial effects on lifespan and aging-associated pathologies. External supplement of PS could increase the oxidative stress resistance and extend the lifespan of nematodes via DAF-16 (Kim and Park, 2020). PS supplement could also improve the memory impairment of the old rodents as well as the human being (Vakhapova et al., 2010; Lee et al., 2015), which is consistent with a crucial role for PS in driving the functional recovery of severed axon and damaged neurons (Hulbert et al., 2007). Moreover, it was shown that compared with elderly unimpaired mice, PS contents in elderly mice with impaired memory are significantly reduced (Wackerlig et al., 2020), further suggesting a strong causal link between the PS contents and the decline of neuronal function during aging. Notably, the principal PS (18:0/22:6) is remarkably increased in the brains of elderly people, consistent with the beneficial role of DHA (22:6) on cognitive function. Both PE and PS containing DHA as acyl chains increase with age (Hancock et al., 2015, 2017; Norris et al., 2015), suggesting that DHA may protect against aging-related neuronal impairment via these PL species.

## Cardiolipin

### Cardiolipin and Aging

Cardiolipin is a symbolic phospholipid of the mitochondrial membrane. CL plays a critical role in many aspects of the mitochondrial function: stabilizes respiratory chain supercomplex, regulates the activity of mitochondrial membrane proteins, and controls essential mitochondrial signaling pathways (Pfeiffer et al., 2003; Dudek, 2017). Due to the special location, CL is closely related to respiration as well as a normal function of the respiratory chain complex, which leads to its association with aging and age-related disease (Shi, 2010; Paradies et al., 2011; Hsu and Shi, 2017). Consistently, CL contents were found to be decreased in the aged nematodes and rodents (Gao et al., 2017; Šmidák et al., 2017), while the contents of CL are notably increased in the adipose tissues of long-lived model Ames dwarf mice compared with normal mice (Darcy et al., 2020), suggesting the idea that increased CLs are beneficial for health and longevity. Indeed, a yeast study shows that perturbation of CL synthesis leads to decreased longevity (Zhou et al., 2009).

### Cardiolipin Peroxidation and Aging

Notably, CLs are highly sensitive to oxidative stress and the peroxidation of CLs can cause damage to the respiratory chain complex, which in turn causes mitochondrial defects and respiratory dysfunction. In line with this, the contents of total CLs were found to decrease, while the peroxidized CLs are significantly increased in the brains of aged rats, which is associated with impaired activity of brain mitochondrial respiratory complex I (Petrosillo et al., 2008). The peroxidation of CLs is influenced by the CL remodeling, a process catalyzed by the acyltransferase (ALCAT1) that synthesizes CLs from lysocardiolipin (Cao et al., 2004). The resynthesized CLs are highly sensitive to oxidative damage by ROS due to the enriched double bonds in poly-unsaturated acyl chains. Thus, the pathological CL remodeling can exacerbate oxidative stress as well as mitochondrial dysfunction (Li et al., 2010, 2012), while the inhibition of CL remodeling may improve the aging-associated functional decline of mitochondria. Indeed, ALCAT1 inhibition or deficiency could improve mitochondrial function, alleviate oxidative stress and prevent disease progression in the context of many aging-related diseases including obesity, Parkinson’s disease, aging-related heart disease, and non-alcoholic fatty liver disease (Li et al., 2010; Liu et al., 2012; Wang et al., 2015; Song et al., 2019). These findings link ALCAT1 to mitochondrial dysfunction and reveal a critical role for CL remodeling in age-related diseases. A balance between the non-peroxidized CLs and peroxidized CLs is crucial for maintaining mitochondrial function and enabling healthy aging.

## Conclusion and Perspective

**FIGURE 1:**
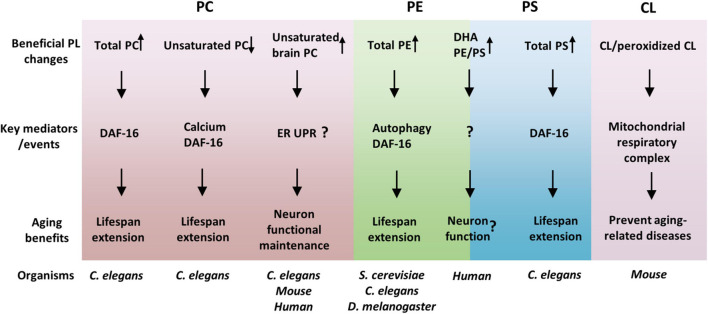
Phospholipids (PLs) regulate aging *via* overlapped but distinct molecular mechanisms. PL species with beneficial effects on healthspan and lifespan were suggested and their key mediators were listed. Among the mediators, DAF-16 seems to be common and important for many PLs effects. Moreover, the functions of organelles, such as the ER and mitochondria, are crucial links between PLs and aging, consistent with PLs as membrane lipids.

As reviewed here, the association between membrane PLs and aging, and the regulation of the aging process and aging-related diseases by PLs have been greatly advanced in recent years. PLs, regulate lifespan and healthspan via overlapped but different cellular and molecular mechanisms (Figure 1). Identification of particular PL species that are associated with lifespan modulation has expanded our knowledge of how metabolic reprogramming affects aging. Nevertheless, these new pieces of knowledge also raise many important and interesting questions, which include, but are not limited to, the following ones: (1) Why are the overall PLs generally decreased with age? It appears that the overall PLs regulate lifespan in ways different from the specific lipids. Whether PLs regulate the aging process via common mediators such as DAF-16/FOXO? If so, how does DAF-16 receive signals from multiple membrane PLs? (2) What are the mechanisms of lifespan regulation by specific PLs, such as unsaturated PCs? It is important to investigate what subcellular compartments the specific PLs function on and what membrane proteins these PLs interact with. (3) How are the contents of PLs regulated during aging? Are there any key transcription factors that control the expressions of PL metabolic enzymes during aging? (4) How does the cell control the homeostasis of PLs? It is unclear how does the cell sense the changes of PL contents and responds/adapts to such metabolic stresses.

The answers to these questions will provide deeper understandings of the function of PL homeostasis in lifespan and healthspan regulation. As the conservation of lipid metabolism across phyla and unique advantages of model organisms in an aging study (such as the short lifespan of *C. elegans* and the tissue similarity to human of rodents), studies combining different model organisms will together help to elucidate the crucial roles and mechanisms of PLs in regulating lifespan and aging-associated diseases.

## Author Contributions

SP conceived the concept of this manuscript. YD, HT, and SP analyzed literatures related to this review topic and wrote the manuscript. All authors contributed to the article and approved the submitted version.

## Conflict of Interest

The authors declare that the research was conducted in the absence of any commercial or financial relationships that could be construed as a potential conflict of interest.

## Publisher’s Note

All claims expressed in this article are solely those of the authors and do not necessarily represent those of their affiliated organizations, or those of the publisher, the editors and the reviewers. Any product that may be evaluated in this article, or claim that may be made by its manufacturer, is not guaranteed or endorsed by the publisher.
